# Mental model for information processing and decision-making in emergency care

**DOI:** 10.1371/journal.pone.0269624

**Published:** 2022-06-09

**Authors:** Modi Al-Moteri

**Affiliations:** Nursing Department, College of Applied Medical Sciences, Taif University, Taif, Saudi Arabia; Texas A&M University, UNITED STATES

## Abstract

**Background:**

Uncertainty and time pressure in emergency departments add a challenge to the rational decision-making process, specifically when encountering a critical patient who requires a prompt response. However, there has been little attempt to develop a mental structure model to understand the thought processes and identify cognitive weaknesses points in nurses’ decision-making. A better understanding can inform changes in both practice learning strategies and decision-making in emergency department. This study aims to better understand how newly employed nurses process information and initiate actions in emergency situations characterized by time constraints and uncertainty.

**Method:**

Participants worked under time pressure and uncertainty to solve a simulated shock case by establishing an assumption of what type of shock the simulated patient might have and its cause. An 8-minute window was available to initiate action. Following the simulation, a retrospective think-aloud interview was conducted.

**Findings:**

Participants’ ability to identify the category of shock was better than their ability to identify the underlaying cause of the shock. This influenced their ability to intervene correctly. Participants’ thinking process in an emergency situation can be organized using ABCDE acronym as follows: (1) **a**wareness of the situation, followed by, an instant (2) generation of **b**eliefs (presumption), (3) **c**ontrolling the **c**onsequence (first-line management action), (4) involvement in **d**eliberate thinking and, finally (5) **e**x**e**cution, actions (second-line management action). The cognitive weakness was mainly noticed during the first-line management action when participants were involved in immediate lifesaving activities.

**Conclusion:**

Classification of the steps involved in decision-making when encountering emergency situations may provide insight into the strengths and weaknesses of the thought process at different stages. Further studies are required.

## Introduction

Uncertainty and time pressure are common and unavoidable in emergency nurses’ practice [1, 2]. These conditions add a challenge to rational thought process, specifically when encountering a critical patient who requires a prompt response [1]. Uncertainty is a "mental state" experienced by nurses when trying to decide between two or more actions [2]. Meanwhile, time constraint is a kind of psychological pressure that add stress on nurses when they have less time available than is necessary to complete a task or obtain an intended care result [3].

Time pressure and uncertainty are widely recognised experienced phenomena that may have substantial negative effect on patient safety [3, 4]. Failure to identify patients who have a serious and potentially life*-*threatening problem is well documented in literature [5]. The problem may be explained in part by poor information processing, causing a delay in responding. Information processing refers to the ability to perceive, interpret and connect “relevant information whilst filtering out unnecessary information” to generate a decision and initiate actions [6].

Information-processing in clinical decision-making is based mainly on two systems of thinking processes, called System 1 and System 2 thinking approaches [7]. System 1 thinking approach is often described as “pattern recognition”, in which a schema in long- term memory is activated by certain cues to form an assumption [7]. It is generated almost instantly without much thinking effort by matching patterns with existing knowledge obtained form similar past situations; this is also known as the “gut feeling”. However, the System 1 Thinking approach does not necessarily produce a correct assumption [8]. Meanwhile, System 2 thinking approach involves “deliberate thinking” and is more “analytical,” and rational [7]. System 2 thinking approach is generated by collecting, searching for additional information. The data are then processed carefully, and consciously [7]. It is, indeed, slower than System 1 Thinking approach and a cognitively demanding process but is more likely to generate better decisions [9]. However, System 2 to System 1 thinking approaches may be used interchangeably to solve problems [7, 9].

Nurses’ practice in emergency settings is characterized by being action-driven rather than analytic-driven [9]. This is because nurses in other clinical settings generally use an analytical method (System 2 Thinking approach) which is an information-seeking method and involves history-taking, physical assessment, and investigations. This method of thinking helps nurses to prove their thinking outcomes and reach a conclusion about the specific situation [10], particularly when the nurses fail to match the patient’s clinical cues with a specific disease classification script [11]. Meanwhile, nurses in emergency settings are required to act immediately in the absence of or having limited information that can be obtained from patient history, physical examination and medical investigations [9].

In spite of the abundance of literature addressing the characteristics of emergency settings [4, 8, 9, 10], there has not been a mental structure model proposed to understand the thought processes of clinicians and to identify particular cognitive weaknesses [6]. A better understanding may inform changes in both practice and decision-making in the emergency department setting.

## Study aim

This study aims to better understand how new graduate nurses process information and initiate actions in emergency situations characterized by time constraints and uncertainty.

## Materials and methods

This is a descriptive exploratory qualitative study design in which retrospective think-aloud interviews were conducted [12] that investigated the underlying thought processes and actions of newly graduated nurses while identifying a type of shock and initiating appropriate actions.

### Setting

Simulation was conducted at the regional hospital in a Simulation Unit (SU). The unit is used as a resource for in-service training of medical clinicians, nurses and other healthcare professionals to improve knowledge, professional and clinical skills for fulfilling various responsibilities. SU provides low and high-fidelity manikins and standardized patients.

### Participants

The simulation session was offered to all newly graduated nurses (n = 25) recently employed at one of the regional hospitals. The simulation session was part of the training and orientation program introduced by the hospital. The program is introduced to the newly employed healthcare providers and included some theoretical lectures, simulation sessions and field training. It is aimed to prepare newly employed healthcare providers for entry into clinical practice by introducing them to the policies and procedures at the workplace (e.g., "vital signs monitoring, waste management, tube insertions, patient assessment, injection administration, intravenous infusion"), new technologies and etc. All the 25 participants were invited to voluntarily join the study. Of those, 12 male nurses agreed to participate.

### Simulation

The SU consisted of three forms of simulation: high fidelity simulators (e.g., SimMan), low fidelity of simulators (e.g. dolls) and a standardized patient. In the current study, the standardized patient was a male trained to portray patient scenarios for the purposes of teaching, training, and evaluation of trainees’ performance. The standardized patient was a certified simulation technician who is in his late 40s and interested in acting and has excellent communication skills. He was selected based on the case requirements which include gender, age, physical appearance, attributes and acting ability and experience. The standardized patient received a two-hour training session preceding the simulation session to ensure that information is retained. He was instructed to depict the case consistently for every trainee. The simulated session was designed to be as realistic as possible to enhance the training experience. The simulated session followed three traditional phases of healthcare simulation: "pre-briefing, simulation, and debriefing".

#### Phase-1: Pre-briefing

Prior to running the ’simulated session’, a pre-briefing phase was conducted. In this phase participants attended a session in which the objectives of the study were explained. Participants were instructed how to complete the simulation and advised that they would be assigned to a 73-year-old diabetic male who had experienced vomiting for four days. Each participant had eight minutes to rapidly assess the patient, record key clinical findings in a chart, and attempt at least one nursing action before moving to the next briefing phase.

#### Phase-2: Simulated session

The ’simulated session’ phase was then conducted. The clinical instructors of the Critical Care Unit designed a scenario of a shock syndrome case that represents a typical clinical emergency situation frequently encountered. Two experts in the field of adult intensive care—intensive care consultants—were invited to review the goals and the content of the designed scenario. Slight modifications were suggested on some of the supporting data associated with the scenario (diagnostic studies results). The experts, then agreed that the case designed was reasonable and reflect the realities of the clinical practice.

There are several categories of shock syndrome—(1) hypovolemic, (2) cardiogenic, and, (3) septic and each results from different cause and has a different medical management. Shock syndrome is defined as the inadequate means of arterial blood pressure to meet the needs of the tissues and body organs [13]. Although the underlying causes of these categories of shock syndrome are different, they have some similarities and differences in clinical presentations. This may create a degree of uncertainty for a nursing clinician and might be mistakenly interpreted [14].

**Fig 1:**
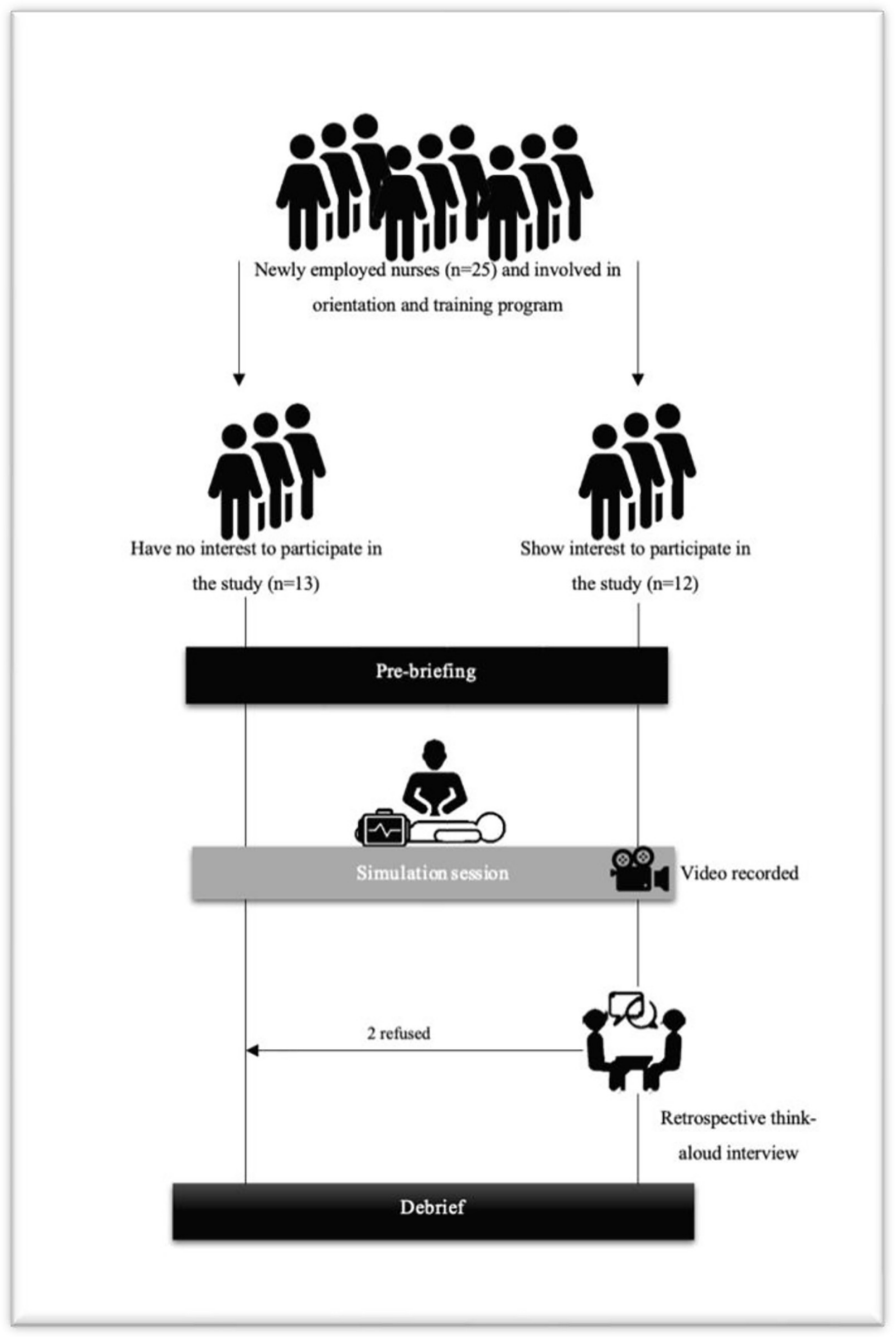
Study recruitment and procedure.

Since this simulated scenario is introduced by a regional hospital for training and orientation purposes, all newly employed nurses (n = 25) are mandated to take part in this program, however, only those (n = 12) who agreed to take part in the current study, were videorecorded (Fig 1).

As the participant entered the room, there is, adult male who is wearing a hospital gown, sitting at a 45-degree angle and showing facial expression of pain. He is making moaning sounds expressing physical suffering, cough, and takes labored breaths. A pulse oximeter probe is placed on a finger, and a nasal cannula is in place; oxygen flow at 2 L/min. The vital signs monitor is turned on. Peripheral IV access is established. A glucose measurement device is placed near the patient. Initial clinical information were:

- Temp: 36.9°C
- Pulse: 134 /minute
- Respirations: 32/minute
- Oxygen saturation: 100%
- Blood pressure: 88/45 mm Hg
- Weight: 70 kg
- Mental status: lethargic; oriented to time, place, and person; cognition intact; answers questions appropriately

At the bedside, a registered nurse taking the role of an emergency department (ED) nurse, hands an ED Note to the participant. During the scenario, the ED nurse provides further scripted information that cannot be portrayed by the simulated patient while staying in role. The ED nurse provides participant the diagnostic studies, if requested by participants.

At the time of the simulation, each participant had eight (8) minutes to assess the standardized patient, take and record key clinical findings (e.g., vital signs, physical appearance, laboratory results) in a chart, and attempt at least one intervention. The time given to participants together with the accessed clinical information was just enough to perform a focused assessment, identify the type of shock the etiology and decide upon action. Since this simulation case could be any of the three shock categories: (1) hypovolemic, (2) cardiogenic, and (3) septic, the equipment necessary to manage each shock syndrome was available and visible on a cart.

The 25 newly employed nurses were divided into two groups: those who agreed to participate (n = 12) and those who had no interest to participate (n = 13). The simulation activity was standardized at 12–15 minutes long and was run 12 to 13 times a day (from 9:00am to 01:00pm) for two consequent days. To prevent participants’ revealing knowledge of the task from their colleagues and peers, the 12 participants who agreed to participate in the study were assigned to be the first to commence the simulated activity.

Immediately after assessing the patient, each participant completed the performance sheet for the simulated patient. Participants noted key observation findings, clicked or circled the suspected shock category and the etiology and the chosen medical and nursing management. For example, the participant could circle “hypovolemic”, “cardiogenic”, or “septic” for the shock category.

#### Phase-3: Interview

Once participants finished their simulated scenario task, they were invited to review the recorded video of their performance and to reflect on their actions. From the 12 participants who showed interest in participating in the study, 10 were interviewed and two refused (Fig 1). A 15–20 min interview was conducted. The participant was again shown a recorded video of his performance and was asked to explain his actions. This retrospective think-aloud interview was conducted by the researcher to promote reflection of participants on their performance with respect to the scenario and to identify the underlying cognitive process they used (S1 Appendix). Questions such as “Help me understand why you do that and this…” and “tell me more about…” was used to reveal the participant’s own thought processes used to interpret the clinical situation. Immediately following the retrospective think-aloud interviews, participants were debriefed to allow them to reflect on the experience, analyse and revealed their emotional states.

#### Phase-4: Debriefing

Once the interviews were completed, participants were gathered for group debriefing. A senior clinical instructor facilitated the debriefing. Participants were encouraged to describe their feelings about the experience. They were also encouraged to state "What went well and what did not go well and why?". They were also asked "What was the main take-home message?".

### Ethical approval

Ethical approval was granted from the Ministry of Health Ethical Committee. Written informed consent was obtained from the willing participants. Participants were informed that their participation was entirely voluntary and that their contribution would enhance the development of clinical practice and training. An Explanatory Statement relating to the study design and purpose were given to the participants. Participants were informed that confidentiality and anonymity would always be maintained and were given the freedom to withdraw from the study at any point in time. Any concerns were addressed. Participants were informed that study results would have no bearing on the formal evaluation of their training program. To maintain privacy, interviews were conducted with the participation of only the interviewer and the interviewee. The study was conducted with the hospital clinical instructors’ involvement following a unit coordinator agreement.

## Data analysis

Ten interviews were transcribed and analysed. For the researcher to understand the cognitive processes underlying the information seeking behaviour in the context of shock syndrome, a hybrid thematic analysis approach was conducted [15]. The approach combined two philosophical reasoning techniques: deductive (a top-down reasoning), and inductive (a bottom-up reasoning) [15]. The inductive analysis phase mainly allows themes to directly develop from the qualitative data. In the current study and during the inductive phase, the researcher read and reread the transcripts to generate a general understanding. Initially, in the inductive phase a total of 74 codes were identified. Saturation was reached after approximately seven interviews. The identified codes were then reviewed, reorganized and similar codes were grouped [16]. A second round of a deductive analysis was initiated to enable an in-depth exploration of data in line with the existing literature. In the deductive analysis phase, the emerged codes were aligned with the findings of the previous studies presented in Al Moteri et al. [4], Al Moteri et al. 2020 [6], Al-Azri [17], Al Moteri [18]. In particular, the theoretical frameworks constructed by Al-Azri [17], Al Moteri [18] have guided the presentation of themes and codes and their interrelationships (Table 1). Themes were then organized using ABCDE acronym for easy use.

**Table 1:** Alignment of emerged codes and themes with existing literature during the deductive phase.

Initial study themes	Al-Azri, ABCDEFGH- Decision-making framework [17]	Al Moteri, [18], Al Moteri et al. [4, 6], Decision-making framework	Final themes ABCDE mental model
Notice something is happening	Awareness, situational	Impression, visual awareness	Awareness, sensory processing
X	Basic life, organ, and limb supportive measures	X	X
Form pre-assumption	X	Recognition	Belief (assumption), generating
Emergencies Immediate action	Control potential life, organ, and limb threats	X	Consequences, controlling
Collate and analyse data [think carefully]	Diagnostics	Deliberate, thinking	Deliberate, thinking
X	X	Refining hypothesis	X
X	Emergency management	X	X
Implement actions	Further care	Execution	Execution, actions
X	Groups of particular interest	X	X
X	Highlights.	X	X

A summary of the findings supplemented with the appropriate descriptive repeated quotes was prepared (S2 Appendix). At this point, five themes were generated and supplemented with the appropriate descriptive repeated quotes. The demographic and performance data were presented in the form of percentage.

### Quality of the data

Several strategies were used to maintain “credibility”, “transferability”, “dependability”, and “conformability” of the data [19]. Transcripts were examined and checked by an external reviewer to ensure “credibility”. For the sake of maintaining “transferability”, participants were invited to judge the end results of data analysis. Ensuring “dependability” was made by careful development and preparation of all the steps of the study, including data collection and analysis. The investigator always kept in mind the aim of the analysis—to investigate the underlying cognitive process of cue recognition. Finally, “conformability” was maintained through using virtual presentation of the data.

## Results

This section may be divided by subheadings. It should provide a concise and precise description of the experimental results, their interpretation, as well as the experimental conclusions that can be drawn.

### Participants’ demographic and performance data

A total of 12 participants agreed to participate and completed the performance sheets. Their ages ranged from 23 to 25 years old, with the mean age being 23.3 years old and the standard deviation being 0.64. They were all male graduated from the same educational institution and their GPAs ranged from 2.6 to 3.4 out of 4, with the mean score of GPA being 2.85 points and the standard deviation being 0.27. Of those 12 participants, 10 completed the interview.

Reviewing the performance sheets of the 12 participants revealed that nine participants (75%) correctly identified the shock category and out of these 9 participants, five (56%) determined the correct etiology. Participants’ failure to identify the etiology has influenced their ability to intervene correctly. Indeed, actions varied considerably. Due to the participants’ characteristic similarity, no association between study subthemes and students’ characteristics was sought.

### Themes

Data from the ten interviews contributed to describe several main mental activities reflecting new graduate nursing students’ actions triggered by uncertainty, namely, (1) **a**wareness of the situation, followed by, a prompt (2) generation of **b**eliefs (presumption), (3) **c**ontrolling the **c**onsequence (first-line management action), (4) involvement in in **d**eliberate thinking and, finally (5) **e**x**e**cution of actions (second-line management action). These themes represent the underlying cognitive processing of the information described by the interviewees.

#### Theme-1: Awareness, sensory processing

When participants met the simulated patient, they instantaneously began to perceive the context. Their perception at this early stage was mainly based on highly visible and easily notable information presented by the patient or in the surrounding environment. In this step, the brain immediately began to process the sensory information obtained from multiple sensory modalities to form general understanding. Emergency nurse’s expectations, skills and knowledge all may contribute to the initial perception of the emergency situation. The instant sensory processing of the contextual information is very important in guiding decision-making when encountering an emergency situation. For instance, some of the participants verbalized how quickly, in a matter of seconds they noticed the appearance and the behavior of the patient:

*“…when I entered the room*, *I immediately noticed how he is in the bed holding his stomach … you see him… he (the patient) is laying on his right side …. curling and holding his stomach…"*

*".. though I didn’t ask him anything yet in that moment but … he is (the patient) frowning and grimacing his face*, *give me a clue that he is in pain……”*

*" … you can hear him moaning …*… *when I entered the room*, *I immediately heard the moaning and when I approached him*, *I found him on the bed and laying on his right side*…*"*

The significance of this instant sensory processing is not reflected adequately in the emergency decision-making process [17]. Typically, decision making in emergency situations has focused on the outcomes rather than the process [17].

#### Theme-2: Belief (assumption), generating

The sensory processing of the contextual information in the previous step is interpreted in a matter of seconds to generate a preliminary clinical assumption (impression). The assumption is formed unconsciously and on the basis of little evidence. It stimulates participants to go beyond the contextual perception of what is going on, as shown in the comment below:

*" …. from his facial expression and body movement…it didn’t take me long to realize he is in pain* *…I can tell he is in great pain…"*

*The way he was holding his abdomen, the grimace on his face*, *… you can see his facial muscles twitching… . I think he is in pain and his pain is getting worse*

#### Theme-3: Consequences, controlling

Participants in the current study attempted to look for leading but critical signs that could reveal immediate risks. The goal of this quick scan is to identify and manage any potential life-threatening conditions. For example, participants ensured optimal breathing and circulation, as shown in the comments below:

*"…he is not choking but he breathes fast…I can tell he is having some serious problem …. a little bit increase of O2 (Oxygen) would do no harm but it may save his life.. you see me too busy giving him O2 mask…I’m thinking …* *he might need a defibrillator.. I should be ready too.."*

*“…. he is vomiting*, *not to say that intake might be decreased… I should immediately put him on a fluid balance if I want to save his life…"*

*“… so like you might go by the BP (blood pressure)*,*… If he is a bit dry, he’s blood pressure might be low…. so the quicker you get cannulas into him, the better end outcome is…"*

*"… . you know if pain is intense*, *he may faint at any moment…. you can see me too busy keeping my eyes on him…"*

It is typical in emergency situations to think about potential threats and intervene promptly. This is done through shortening the thinking process rather than a detailed evaluation. Mental shortcuts are considered useful and even necessary in emergency situations under time constraint and uncertainty. Mental shortcuts offer rapid assessment and permit immediate action to safe patient’s life. However, this fast thinking does not always lead to correct actions and outcomes. For example, the assumption of bleeding might be made based on signs such as low blood pressure and the high pulse rate, as follows:

*“…from a lower blood pressure and a high pulse rate*… *I can tell there’s a bleed going on there……I should administer blood or call for help…”*

*“.. his (the patient) blood pressure is dropping… he’s losing blood*; *it is life threatening problem,.…I should get it up (blood pressure) with legs up”*

#### Theme-4: Deliberate, thinking

Once immediate risks and threats have been controlled and managed, participants may start to devise a workable clinical assessment through an analytical process. Participants in this stage tend to arrange, analyse and relate clinical information, form some queries that attempt to test the assumption by looking for information that confirm or, alternatively, reject it. See the comments below:

*“…I checked ECG*, *I wanted to make sure that it wasn’t cardiac problem…”*

*“BP (blood pressure) low,…. I thought well*, *I’d better check his temperature because if he got a rupture appendix a bit of a temperature that might be going up….but the temperature was normal”*

*“I thought*, *well if the urine is dark that’s give me clue that he (patient) is dehydrated …. and obviously the BP (blood pressure) is low.…"*

*“…I auscultate the chest….you know it is common to have lung disease when you are old*.. *but the lung was clear again and the CXR (chest x-ray) was also clear…"*

#### Theme-5: Execution, actions

Based on the outcomes of the deliberate thinking, an overall plan that includes actions to manage the patient problem is produced. This step does not oppose the action initiated earlier to safe the patient’s life (**C**), nevertheless it helps emergency nurse to have holistic view to manage the case. See the comments below:

*“… so you go by dehydration*… . *you can see me giving him antiemetic and monitoring fluid balance…"*

## Discussion

This study explored the thought processes of 10 newly graduated nurses while they managed patients with unknown shock syndromes using retrospective think-aloud interviews. To our knowledge no previous studies have explored the thought process of nurses under time pressure and uncertainty in relation to emergency clinical problem. This study gives new insights into how the inherent uncertainty in patients with unknown problems is managed by nurses in emergency care. For nurses, working in emergency settings in hospitals is always "stressful, time sensitive, life-critical, and information poor and loaded" [6]. Making an optimal decision under such conditions is difficult and sometimes challenging [5]. How health providers use their cognitive and attention resources in emergency situations is sometimes overlooked area of study [17]. This study dissects the mental activities of nurses in emergency care into five steps. These steps might be used to represent the thought process of nurses during emergency decision making when encountering a critical patient who requires prompt response.

**Fig 2:**
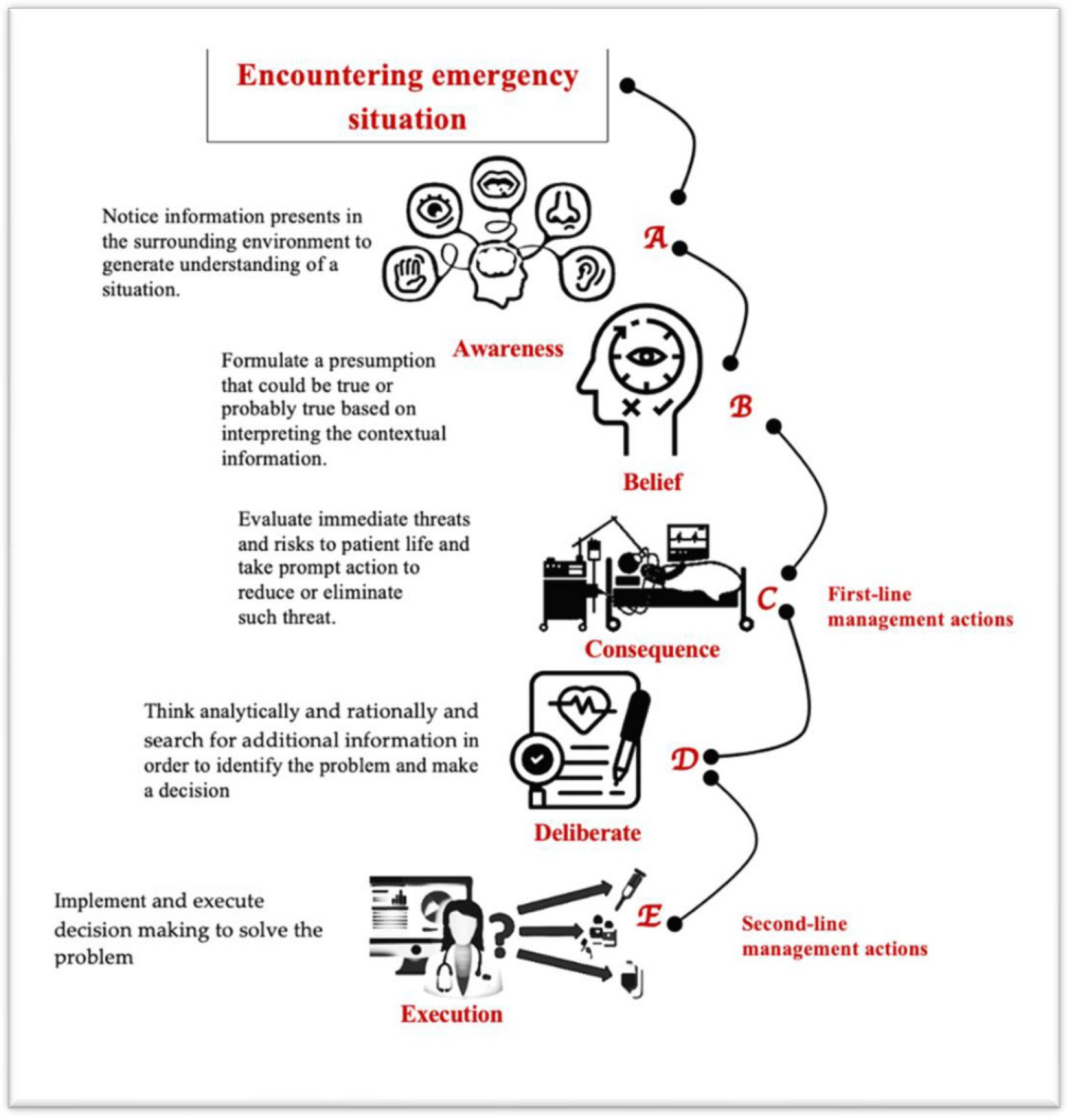
A proposed theoretical framework.

A mental model (Fig 2) was proposed as a summary of the research findings. It illustrates the process of decision-making when encountering an emergency situation with limited information. The proposed mental model steps were arranged in an alphabetical order from A to E for easy use and recall: (**A**) awareness of the situation by sensory processing of the contextual information present in the patient and environment. Evidences have suggested that being aware of the situation increases the likelihood of a “good” decision [20], specifically in urgent situations that demand rapid decision-making [21]. Awareness can be achieved through many sensory channels (visual, auditory and touch) [21, 22]. In the current study participants visually observed the patients’ facial expression and the position of the patient on the bed and heard the voice the patient produced. Once awareness is maintained, an instant (**B**) belief (presumption) about the situation is generated through interpreting contextual issues. In emergency situations where uncertainty is high, healthcare providers may rely on highly visual and readily available information to make a decision [23]. In the current study, the nurses assume that the patient has pain from interpreting the patient’s facial expression and behavior. Such an assumption is a typical System 1 Thinking approach and is generated almost immediately upon encountering the patient [5]. This is followed by close observation and a search for critical signs that may indicate the presence of threats on the patient’s life in an attempt to (**C**) control the consequence by initiating immediate lifesaving actions. This is in line with what was reported in the literature [17]. Nevertheless, step (C) may not be correct under uncertainty and time pressure. Indeed, in the current study inappropriate actions to prevent further deterioration of the patient, were noticed. Although it was not the focus of the current study to investigate why it was more likely for participants to make errors in (**C**) step under time pressure, researchers reported that time pressure tends to increase the perception of the difficulty of the task [24, 25]. The (**C**) step however, represents a connection point between System 1 Thinking approach—step (**B**), and System 2 Thinking approach in the next step (**D**) which involves a deliberate thinking. Step (**D**) involves analyzing and investigating clinical information for optimal decision-making. Finally, in (**E**) execution, actions are initiated to start intervention or to continue observing and monitoring the patient.

The proposed conceptual mental model may contribute to the development of certain clinical reasoning skills useful to develop in emergency practice, particularly, those used in emergency settings. In addition, the practical classification of the thought processes of nurses during emergency decision-making provided additional insight into the cognitive weaknesses in the process in which lapses and errors may occur [6]. Hence, to devise practical training to strengthen the weak areas of thinking may be helpful. An inherent issue in emergency setting work is its chaotic nature [17]. The best approach to manage a disordered context is by establishing order [17]. The proposed conceptual mental model addresses this issue by organizing emergency nurses’ activities (A, B), prompt acts (C) which represent the first-line management actions and involve a triaging process in order to identify the most appropriate management-action to initiate an immediate lifesaving action; then finally responses (D, E) and this includes the second-line management actions and involves analyzing and planning for further care for the patient if required. These stages may create a more organized working environment.

## Implication

Study findings bear several implications. Showing how nurses think in emergency situations, where there is great time pressure and uncertainty, is very important. Indeed, knowing nurses thinking process not only helps to improve patients’ outcomes, it also supports the improvement of the emergency nurses at all levels. Teaching the mental model to newly employed nurses working in emergency departments and critical care units will contribute to their understanding of their own mental processes and limitations. In addition to newly employed nurses, this model could also have implications for anyone practicing emergency care such as paramedics and general nurse practitioners in rural clinics. The theoretical mental model gives clinical educators a practical starting point to develop training and educational methods that elaborate each thinking stage separately, hence, identifying cognitive weaknesses and training nurses to avoid them. This is extremely important for the newly employed nurses who initially require extra support as they obtain their practical experience and combine it with their existing theoretical knowledge. It is also important in assisting experienced nurses in their practice by enhancing self-awareness of their own mental processes and limitations. Highlighting the importance of the theoretical mental model to nurses in emergency situations to support their thinking process may encourage the field to implement formal follow up processes enabling nurses to review their thinking process outcomes. This would contribute to learning opportunities by identifying areas for improvement and gaps in clinical decision making. The model may also be used to develop clinical decision-making tools tailored to the needs of the emergency practice. The study findings have also raised an opportunity for further investigation into the underlying mental processes of nurses’ decision-making under time pressure and uncertainty.

## Limitations

Although this study adds valuable information to the body of knowledge, there are several limitations. Firstly, the homogeneity of the sample interferes with the generalizability of the results. Studies involving different nurses from different geographic populations is recommended. Secondly, factors such as confidence and mental capabilities have been found to influence the cognitive process and were not investigated in this study. These factors should be considered in future research. The sample also has included recently employed novice nurses who may lack of adequate experience. Experience is seen to be a critical factor to enhance an individual’s own thought process and decision-making. More studies are recommended in this area of investigation. Finally, the focus of the current study was only to better understand how new graduate nurses process information and initiate actions in an emergency situation characterized by time constraint and uncertainty; more studies are required to investigate the contributing factors underlying errors in initiating actions.

## Conclusions

The study provides new insights into decision-making and thinking processes under conditions of time pressure and uncertainty. Newly employed nurses apply System-1 and System-2 thought processes when encountered with emergency situations. System-1 thinking approach was the influencing factor in how decisions were made. Newly employed nurses have relied on the easily accessible contextual information to form an initial impression. They displayed their ability to use minimum information to identify life threatening issues and modulated their actions accordingly. They are more likely to make errors as they decide upon the actions required to perform life-saving measures. The implications of this proposed mental model for emergency nursing practice are: decision-making structure guidance; better cognitive performance in emergency settings in relation to decision-making; and encouragement in the implementation of formal follow up, thus supporting continued improvement in practice to better thought process outcomes. However, the suggested approach requires further studies.

## Supporting information

S1 Checklist COREQ (COnsolidated criteria for REporting Qualitative research) checklist. (PDF)

S1 Appendix Retrospective think-aloud interview protocol. (DOCX)

S2 Appendix (XLSX)

S1 File Simulation case. (DOCX)

S2 File Trainee answer sheet. (DOCX)

S3 File (XLSX)

## References

[1] Franklin A, Liu Y, Li Z, Nguyen V, Johnson TR, Robinson D, et al. Opportunistic decision making and complexity in emergency care. Journal of biomedical informatics. 2011 Jun 1;44(3):469–76. doi: 10.1016/j.jbi.2011.04.001 21511054

[2] Cranley L, Doran DM, Tourangeau AE, Kushniruk A, Nagle L. Nurses’ Uncertainty in Decision‐Making: A Literature Review. Worldviews on Evidence‐Based Nursing. 2009 Mar;6(1):3–15. doi: 10.1111/j.1741-6787.2008.00138.x 19302543

[3] Vinckx MA, Bossuyt I, de Casterlé BD. Understanding the complexity of working under time pressure in oncology nursing: A grounded theory study. International journal of nursing studies. 2018 Nov 1;87:60–8. doi: 10.1016/j.ijnurstu.2018.07.010 30055374

[4] Al-Moteri MO, Symmons M, Cooper S, Plummer V. Inattentional blindness and pattern-matching failure: The case of failure to recognize clinical cues. Applied ergonomics. 2018 Nov 1;73:174–82. doi: 10.1016/j.apergo.2018.07.001 30098633

[5] Al-Moteri M, Plummer V, Cooper S, Symmons M. Clinical deterioration of ward patients in the presence of antecedents: A systematic review and narrative synthesis. Australian Critical Care. 2019 Sep 1;32(5):411–20. doi: 10.1016/j.aucc.2018.06.004 30025983

[6] Al-Moteri M, Cooper S, Symmons M, Plummer V. Nurses’ cognitive and perceptual bias in the identification of clinical deterioration cues. Australian Critical Care. 2020 Jul 1;33(4):333–42. doi: 10.1016/j.aucc.2019.08.006 31615698

[7] Tay SW, Ryan P, Ryan CA. Systems 1 and 2 thinking processes and cognitive reflection testing in medical students. Canadian medical education journal. 2016 Oct;7(2):e97. 28344696 PMC5344059

[8] Burbach BE, Thompson SA. Cue recognition by undergraduate nursing students: An integrative review. Journal of Nursing Education. 2014 Sep 1;53(9):S73–81. 25102133 10.3928/01484834-20140806-07

[9] Croskerry P. Clinical decision making in emergency medicine. Clinical Reasoning in the Health Professions. 4th ed. New York, NY: Elsevier. 2018 Oct 15:285–94.

[10] Rababa M, Al-Rawashdeh S. Critical care nurses’ critical thinking and decision making related to pain management. Intensive and Critical Care Nursing. 2021 Apr 1;63:103000. doi: 10.1016/j.iccn.2020.103000 33376039

[11] Croskerry P. ED cognition: any decision by anyone at any time. Canadian Journal of Emergency Medicine. 2014 Jan;16(1):13–9. doi: 10.2310/8000.2013.131053 24423996

[12] Charters E. The use of think-aloud methods in qualitative research an introduction to think-aloud methods. Brock Education Journal. 2003 Jul 1;12(2).

[13] Standl T, Annecke T, Cascorbi I, Heller AR, Sabashnikov A, Teske W. The nomenclature, definition and distinction of types of shock. Deutsches Ärzteblatt International. 2018 Nov;115(45):757. doi: 10.3238/arztebl.2018.0757 30573009 PMC6323133

[14] Lammers R, Pazderka P, Sheakley M. A Multipatient Simulation Session: Evaluation of Six Simulated Patients with Different Shock Syndromes. MedEdPORTAL. 2017 Jun 7;13:10591. doi: 10.15766/mep_2374-8265.10591 30800793 PMC6354717

[15] Swain J. A hybrid approach to thematic analysis in qualitative research: Using a practical example. SAGE Publications Ltd; 2018.

[16] Sandelowski M. Whatever happened to qualitative description?. Research in nursing & health. 2000 Aug;23(4):334–40. doi: 10.1002/1098-240x(200008)23:4&lt;334::aid-nur9&gt;3.0.co;2-g 10940958

[17] Al-Azri NH. How to think like an emergency care provider: a conceptual mental model for decision making in emergency care. International Journal of Emergency Medicine. 2020 Dec;13(1):1–9. 32299358 10.1186/s12245-020-00274-0 PMC7164351

[18] Moteri MO. Investigating failure to recognize clinical deterioration cues among less and more experienced nurse participants. *Doctoral dissertation*, 2016. Monash University.

[19] Lincoln YS, Lynham SA, Guba EG. Paradigmatic controversies, contradictions, and emerging confluences, revisited. The Sage handbook of qualitative research. 2011 Apr 27;4(2):97–128.

[20] Levin S, Sauer L, Kelen G, Kirsch T, Pham J, Desai S, et al. Situation awareness in emergency medicine. IIE Transactions on Healthcare Systems Engineering. 2012 Apr 1;2(2):172–80.

[21] Blandford A, Wong BW. Situation awareness in emergency medical dispatch. International journal of human-computer studies. 2004 Oct 1;61(4):421–52.

[22] Lee L, King G, Freeman T, Eva KW. Situational cues surrounding family physicians seeking external resources while self-monitoring in practice. Advances in Health Sciences Education. 2019 Oct;24(4):783–96. doi: 10.1007/s10459-019-09898-1 31123847

[23] Beglinger B, Rohacek M, Ackermann S, Hertwig R, Karakoumis-Ilsemann J, Boutellier S, et al. Physician’s first clinical impression of emergency department patients with nonspecific complaints is associated with morbidity and mortality. Medicine. 2015 Feb;94(7). doi: 10.1097/MD.0000000000000374 25700307 PMC4554174

[24] Orique SB, Despins L, Wakefield BJ, Erdelez S, Vogelsmeier A. Perception of clinical deterioration cues among medical‐surgical nurses. Journal of Advanced Nursing. 2019 Nov;75(11):2627–37. doi: 10.1111/jan.14038 31012138

[25] Van der Vegt A, Zuccon G, Koopman B, Deacon A. How searching under time pressure impacts clinical decision making. Journal of the Medical Library Association: JMLA. 2020 Oct 1;108(4):564. doi: 10.5195/jmla.2020.915 33013213 PMC7524617

