# Supplementary material for: Mental model for information processing and decision-making in emergency care
Source: PLoS One. 2022 Jun 9;17(6):e0269624. doi: 10.1371/journal.pone.0269624 (PMC9182258; doi:10.1371/journal.pone.0269624)
Supplement: S1 Appendix — (DOCX) [file pone.0269624.s002.docx]

**Retrospective think-aloud interview protocol**

The interview requires you to think aloud as you watch your recorded video of your performance to reflect on your actions. "Think aloud" means you tell me everything you think about as watch you your performance.

When I ask you to tell me everything, I really mean every single thought that comes into your mind from the moment you encountered the patient to the moment they told you to leave the simulation scenario.

It may be unusual to watch your performance and try to remember what thought comes in your mind that moment but try to remember all thoughts. Sometimes, you may find it hard to remember your thoughts —if so, this is okay but please try hard to tell me what your thoughts were as much as you possibly can.

Please note, this study is highly exploratory. My intention is not to evaluate or rate your thinking, but to explore your thoughts as you —and other participants— manage the clinical situation.

Let me start with you:

What the comes in your mind in the moment you entered the room and encountered the patient? Now, please tell me everything you think about as you encounter the patient.
