# Supplementary material for: Mental model for information processing and decision-making in emergency care
Source: PLoS One. 2022 Jun 9;17(6):e0269624. doi: 10.1371/journal.pone.0269624 (PMC9182258; doi:10.1371/journal.pone.0269624)
Supplement: S1 File — (DOCX) [file pone.0269624.s004.docx]

Simulation Case

| Evaluation of Simulated Patients with Shock Syndromes. | |
| --- | --- |
| Case description | A 73-year-old diabetic male patient. Present to emergency department with uncontrolled vomiting for four days. |

| Setting the scene |
| --- |
| An adult male patient who is on his 70s wearing a hospital gown, sitting at a 45-degree angle.   - The patient is and showing facial expression of pain and making moaning sounds expressing physical suffering and takes laboured breaths. - A pulse oximeter probe is placed on a finger, and a nasal cannula is in place; oxygen flow at 2 L/min. - The vital signs monitor is turned on. - Peripheral IV access is established. - A glucose measurement device is placed near the patient. - A preformatted evaluation matrix is on a clipboard.   The patient script, if requested: "Hello, I'm tired, vomiting for 4 days. I'm Type I diabetic and has been out of insulin for 1 week  At the bedside, a registered nurse taking the role of an emergency department (ED) nurse, hands an ED Note to the trainee.   - During the scenario, the ED nurse provides further scripted information and will describe physical findings that cannot be portrayed by the patient while staying in role. The ED nurse script is: "Hello, this patient come to ED complaints of vomiting and stomach pain … he has rapid pulse and his blood pressure is low. |

| Instruction |
| --- |
| Trainee has been informed that he is assigned to the patient and have eight minutes to assess the patient, record key clinical findings in a chart and attempt at least one nursing actions. |

| Patient clinical presentation | |
| --- | --- |
| Vital signs | - Temp: 36.9^o^ C - Pulse: 134 /minute - Blood pressure: 88/45 mm Hg - Respirations: 32/minute - Weight: 70 kg - Oxygen saturation: 100% |
| Medical history | - Diabetes mellitus type 1, on Lantus, Novolog - Not known surgical, allergies and family history |
| Physical Examination | |
| General | lethargic |
| HEENT | dry oral mucous membranes |
| Neck | no jugular venous distention; trachea midline |
| Lungs | tachypnea; clear to auscultation |
| Cardiovascular | tachycardia |
| Abdomen | non-tender |
| Neurological | normal |
| Skin | dry; decreased turgor; pale |
| GU | normal |
| Mental status | Lethargic; oriented to time, place, and person; cognition intact; answers questions appropriately |

| **Treatment orders options** |
| --- |
| An array of treatment options associated with doctor order is visible on a cart, including:   1. Administer Epinephrine (Vasopressor) IM injection—0.3 mg. 2. Administer Amiodarone (anti-arrhythmic agent) IV bolus—300 mg 3. Administer Metoclopramide10mg IV 4. Administer Calcium channel blocker—IV infusion—20 mg 5. Administer Broad-spectrum-antibiotic IV infusion. 6. Administer Glucagon IV bolus—5 mg. 7. Administer Insulin (Intravenously) 8. Administer Morphine 5 mg/mL IM 9. Administer Sodium Chloride — 1 Litre 10. Administer 5% Dextrose in water— 1 Litre 11. Administer packed red blood cells—1 unit. 12. Provide non-rebreather mask (95% FIO2) 13. Provide Hudson Mask (50%FIO2) 14. Prepare airway equipment 15. Prepare defibrillator (Synchronized cardioversion—200 joules) 16. Insert an 18-gauge IV cannula 17. Prepare ECG equipment 18. Change position to upright 19. Change position to flat 20. Change position to left lateral decubitus position. 21. Change position to Trendelenburg position |

| Emergency Department Triage Note |
| --- |
| Information in ED Note:  Patient name: Abdullah Mohammed  Age: 73 y/o; male  Chief complaint: Vomiting x 4 days  Medical history: Diabetes mellitus, type 1  Medications: Lantus, Novolog insulin  Surgical history: None  Family history: None  **Allergies:** Not Known  Nurse’s Evaluation: Dry Skin  Treatment initiated: Nasal oxygen cannula |

Diagnostic studies provided by ED nurse, if requested

1. Complete Blood Count

| Parameter | Value | Reference |
| --- | --- | --- |
| White blood cells | 14.0 x 10^9^ cells/mcL | 3.5-10.5 x 10^9^ cells/mcL |
| Red blood cells | 5.79 x 10^12^ cells/mcL | 5.7-8.8 x 10^12^ cells/mcL |
| Hemoglobin | 13.0 g/dL | 13.5-17.5 g/dL |
| Hematocrit | 38.5% | 38.8-50% |
| Platelets: | 236,000 x10^3^ mcL | 150-450 x10^3^ mcL |

1. Metabolic Panel

| Parameter | Value | Reference |
| --- | --- | --- |
| Na | 132 mEq/L | 135-144 mEq/L |
| K | 5.7 mEq/L | 3.7-5.2 mEq/L |
| Cl | 93 mEq/L | 97-108 mEq/L |
| CO2 | 21 mEq/L | 22-29 mEq/L |
| BUN | 28 mg/dL | 7-20 mg/dL |
| Cr | 1.9 mg/dL | 0.8-1.4 mg/dL |
| Glucose | 822 mg/dL | 64-128 mg/dL |
| Ca | 8.9 mg/dL | 8.5-10.6 mg/dL |

| Parameter | Value | Reference |
| --- | --- | --- |
| pH | 7.43 | 7.35-7.45 |
| Lactate | 4.1 mEq/L | 0.5-2.2 mEq/L |

Radiology report

Chest Radiograph (Plain Film; PA & lateral views): Normal


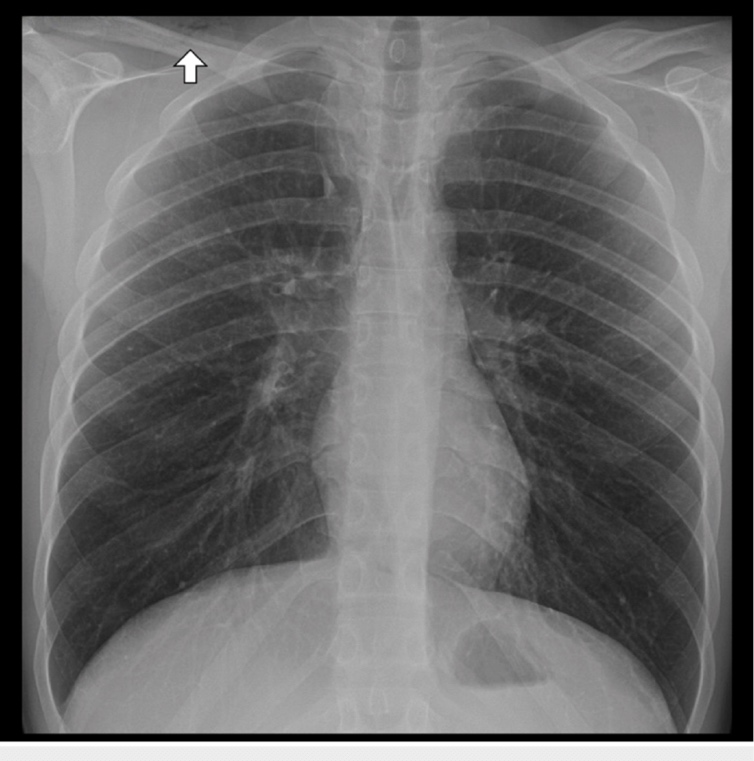


Provided by ED nurse, if requested

12-lead ECG

Sinus tachycardia


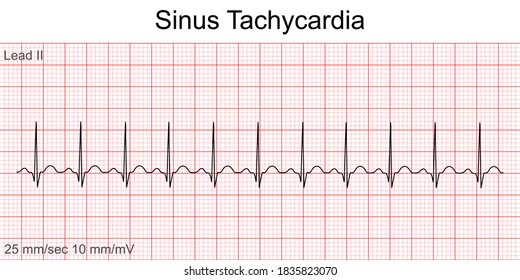


Provided by ED nurse, if requested
