# Supplementary material for: Mental model for information processing and decision-making in emergency care
Source: PLoS One. 2022 Jun 9;17(6):e0269624. doi: 10.1371/journal.pone.0269624 (PMC9182258; doi:10.1371/journal.pone.0269624)
Supplement: S2 File — (DOCX) [file pone.0269624.s005.docx]

**Trainee Answer Sheet**

**Suspected Shock Category**

- Hypovolemic
- Cardiogenic
- Septic

**Suspected Etiology**

Hypovolemic

- Bleeding
- Dehydration

Cardiogenic

- Myocardial Infarction
- Angina

Aseptic

- Acute Appendicitis
- Acute Respiratory Infection

**Action required**

- Administer Epinephrine (Vasopressor) IM injection—0.3 mg.
- Administer Amiodarone (anti-arrhythmic agent) IV bolus—300 mg
- Administer Metoclopramide10mg IV
- Administer Calcium channel blocker—IV infusion—20 mg
- Administer Broad-spectrum-antibiotic IV infusion.
- Administer Glucagon IV bolus—5 mg.
- Administer Insulin (Intravenously)
- Administer Morphine 5 mg/mL IM
- Administer Sodium Chloride — 1000 ML
- Administer 5% Dextrose in water— 1000 ML
- Administer packed red blood cells—1 unit.
- Provide non-rebreather mask (95% FIO2)
- Provide Hudson Mask (50%FIO2)
- Prepare airway equipment
- Prepare defibrillator (Synchronized cardioversion—200 joules)
- Insert an 18-gauge IV cannula
- Prepare ECG equipment
- Change position to upright
- Change position to flat
- Change position to left lateral decubitus position.
- Change position to Trendelenburg position
